# The effects of traditional healing on HIV and AIDS management: An ethnographic study

**DOI:** 10.4102/safp.v64i1.5559

**Published:** 2022-08-19

**Authors:** Avhatakali A. Ndou-Mammbona

**Affiliations:** 1 Department of Health Studies, Faculty of Humanities, University of South Africa, Pretoria, South Africa

**Keywords:** culture, ethnography, healing, impact, management, traditional healer

## Abstract

**Background:**

This article presents the effects of traditional healing on the management of human immunodeficiency virus (HIV) and acquired immune deficiency syndrome (AIDS) in the Vhembe district, South Africa. The Vhembe district is one of the rural districts in Limpopo Province, South Africa, in which traditional healers are used as the first point of consultation for most ailments, regardless of their causes.

**Methods:**

This ethnographic study was based on Leininger’s theory of culture care diversity and universality. It was carried out in selected villages in the Vhembe district. Observation and interviews with 15 purposively selected key informants, who are traditional healers, were used to collect data. Interviews were tape-recorded and field notes were also taken. The data were analysed using the ethnographic content analysis method.

**Results:**

The results suggest that traditional healing has both negative and positive effects on HIV and AIDS management. The positive effects are the effective treatment of some opportunistic infections, such as diarrhoea, skin lesions and childhood diseases. Negative effects, however, include incisions to let the ‘dirty blood flow out’ and inducing of vomiting and diarrhoea, which may lead to anaemia, dehydration and electrolyte imbalances. Some traditional healers are of the view that HIV does not exist and that people either have an ancestral calling or are bewitched. Even though their claims have not been scientifically proven, some traditional healers stated that they can heal HIV and AIDS.

**Conclusion:**

The research brings insight as to whether Vhavenda traditional healing has a favourable or unfavourable impact on HIV and AIDS management. Using Leininger’s steps for adaptation for culture care diversity and universality will help with re-Africanisation of HIV management. The researcher recommend the modification of practices with a high risk of HIV infection to reduce this risk, whilst also supporting the continuation of beneficial practices that reduce HIV mortality, such as diarrhoea management.

## Introduction

This article presents the role of traditional healing methods in the treatment of human immunodeficiency virus (HIV) in a South African district of Vhembe. In this study, management refers to the process of treatment, and care related to HIV and acquired immune deficiency syndrome (AIDS). According to epidemiological estimates from the Joint United Nations Programme on HIV and AIDS (UNAIDS),^1^ 37.7 million people worldwide are living with HIV. Human immunodeficiency virus is still a major problem worldwide, with 680 000 people dying from AIDS-related deaths in 2021.^1^ The Vhavenda people are deeply rooted in their culture and believe that they are suffering from an ancestral calling and witchcraft, not HIV, and their first port of call is mostly traditional healers. In rural South Africa, traditional healers provide physical and psychological services to > 80% of the population.^2^ People seek hospital care only when they are seriously ill. This means that medical care is not sought early in the course of the disease, and this negatively impacts the treatment of HIV and AIDS. Using traditional healers has been associated with delays in HIV diagnoses and also lead to reduced antiretroviral (ARV) adherence amongst people living with HIV.^3^ Traditional healers believe that there is no HIV, and this impacts negatively on the management of HIV as they end up influencing their clients to believe that they can heal them. Contrary to these findings, is the study^4^ conducted in Bangladesh, which revealed that the Kabiraji traditional healers in Bhabanipur and Jobra villages believe that they have healed many diseases, including HIV. This delays patient testing and treatment and compromises case management if they do turn out to have contracted HIV. Other researchers^4^ are also of the view that traditional healers are not officially recognised at all by the governments in many countries and that they nonetheless offer treatment outside the formal healthcare structures. However, leaving traditional healers on the sidelines can have serious consequences because of the high level of dependence that people have on them, leading to delays in testing and the taking of antiretrovirals.

Traditional healers are also exposed to bodily fluids, including blood, when making incisions in the treatment of their clients, and this exposes them to HIV infection. Evidence from a study^2^ in South Africa established that traditional healers are exposed to patients’ blood and bodily fluids and that this puts them at risk of contracting HIV whilst treating their clients.

A lack of training as well as the improper use and donning of personal protective equipment (PPE) increase the risk of traditional healers contracting HIV when in contact with bodily fluids whilst treating patients living with HIV. This statement was attested by other researchers,^2^ who stated that the low ratio of traditional healers trained on the use of PPE (gloves, safety glasses, facemasks, respirators) put them at risk of contracting and spreading HIV. In South Africa, one of the sub-Saharan countries where HIV prevalence has increased sharply from 13.1% in 2018 to 13.7% in 2021, and where 8.2% of people were estimated to be living with HIV in 2021,^5^ the role of traditional healing is paramount in addressing HIV and reducing AIDS-related deaths. These health gaps need to be addressed by ensuring that traditional healers and doctors work together so that people do not delay seeking healthcare and traditional healers do not feel that they are left out in the treatment of HIV and AIDS. Traditional healers should be trained in the treatment of HIV and AIDS, use and wearing of PPE and infection prevention control so that they are able to treat people with HIV without compromising their health and delaying their treatment. Both the Ministry of Health and traditional healers should work together to achieve proper management of HIV and AIDS by both health institutions and traditional healers, with neither misleading the other due to a lack of knowledge on the part of the traditional healers and their clients. The study^6^ conducted in rural Tanzania found that denial amongst those infected with HIV is an issue and that there is a lack of knowledge when it comes to the management of HIV by traditional healers, as they believe that they can heal HIV.

## Methods

### Design

The researcher chose an ethnographic design for this study. An ethnographic study is a qualitative research design that is used to describe and characterise the behaviour and identity of a group or culture.^7^ This design was chosen because the researcher wanted to gain insight into Vhavenda cultural practices related to traditional healing in the management of HIV and AIDS. The researcher felt that the ethnographic design was more appropriate because it allowed the researcher to interact with the traditional healers to better understand the healing processes involved in the management of HIV and AIDS by the Vhavenda people in Vhembe district.

### Study setting

The study was conducted in one of the rural districts of South Africa, Vhembe district in Limpopo province. In Vhembe district, 52 408 (10.1%) people were living with HIV and AIDS; the prevalence rate was 5.4% and 39 800 were taking ARVs.^8^

### Population and sampling

The population was 15 purposively selected traditional healers who met the selection criteria. Included in the study were traditional healers aged 35 years and above who agreed to participate in the study. The study excluded traditional healers who were not willing to participate in the study and those who were either not physically well or not available during the collection of data. Sample size was determined by data saturation.^9^

### Data collection

Semistructured interviews and observation guides were developed prior to beginning of data collection, with these being based on the literature review, study objectives and research questions. A.A.N-M. collected data between August 2019 and December 2019 in the form of individual semistructured interviews and observation instruments that simultaneously followed ethnographic design principles. Interviews commenced with the question, ‘Can you kindly share with me how you heal people with HIV?’ Following the participant’s response, probes and prompts were used to elicit more detailed information from participants. All interviews were recorded and lasted approximately 45–60 min each. Concurrently, participants were observed during traditional healing. Field notes were also taken to describe nonverbal cues during the interviews and observations.

### Analysis of data

The collected data were manually analysed using ethnographic qualitative content analysis.^10^ The researcher used audio-recordings, along with field notes and observation tools, for verbatim transcriptions of the recordings to increase the accuracy of the data collected. All transcripts and field notes were read and reread to code and categorise themes. Coding is the starting point for most forms of qualitative data analysis and it means that the coded data are not presented in their original format but are interpreted by the researcher.^11^ The researcher then looks for relationships amongst developing themes, followed by grouping themes that are comparable to higher-level themes. The researcher creates a main theme table with superordinate themes, themes, subthemes and quotes from the transcript. Samples of the recorded interviews, field notes and observation instruments were transcribed and analysed by an independent researcher and coder (who is an expert in qualitative data) to ensure consistency. Coding reliability was ensured by having another person code the same data and then checked for agreement.^12^ The researcher ensured the trustworthiness of the data by returning to all participants and asking them if the thorough explanation reflected their knowledge and experience. The researcher then reviewed the description of the data to ensure there were no uncertainties, ambiguities or misunderstandings. The researcher conducted a validity check to ensure that the themes were relevant to the data collected.^13^ Following this process described, the researcher developed a single master table of themes from the individual transcripts. The following superordinate themes emerged from the data analysis: positive aspects of traditional healing in the management of HIV and AIDS and negative aspects of traditional healing in the management of HIV and AIDS. The superordinate themes have five themes, namely (1) treatment of opportunistic infections, (2) instructions given to patients during treatment, (3) adherence to ethics, (4) promise of cure and (5) denial of the presence of HIV by traditional healers.

### Ethical considerations

The Department of Health Studies and the Research Ethics Committee of the University of South Africa granted the researcher ethical clearance for the study (ref. no. HSHDC/902/2019). Permission was also obtained from Vhembe House of Traditional Leaders (Vhavenda Kingship Council) which reports to the Department of Co-operative Governance, Human Settlement and Traditional Affairs.

Before starting the study, all the key informants gave their verbal consent. Key informants were informed that the participation was voluntary and that the interviews would be audio-recorded as part of the consent process. Key informants were also told that they might decline or stop participating at any moment if they so desired. Key informants were promised, both verbally and in writing, that their information would be kept private and that the results would be disclosed in such a way that they could not be identified. The transcripts were stripped of any identities, pseudonyms were used to increase anonymity and the data was securely stored on a password-protected computer. The study participants were given a full information sheet outlining the study’s contents, and they granted informed consent after processing all the pertinent information.

## Findings

### Participants’ demographic data

The names of the key respondents were not provided in the study, but pseudonyms were used. The researcher provided the demographics to ensure transferability and comparability of the results to other situations comparable to the setting studied.^14^ Information included key informants’ pseudonyms, ages, gender, traditional practices, and years of practice. The information is provided so that readers can understand the sources of the data. The demographic results of the study show that about 60% of the key informants were male and 40% were female. One observation was made concurrently with the interviews to support the practices of traditional healers, namely the process of traditional healing when removing *goni* from a baby.

See Table 1 for a table of parent themes, topics and subtopics. Excerpts from key informants are presented in indented text, followed by pseudonyms of key informants to support the themes.

**TABLE 1:** Summary of the results.

Superordinate themes	Themes	Subthemes
Positive aspects of traditional healing in the management of HIV and AIDS	Treatment of opportunistic infections	Medicines for skin lesions
		Stopping of vomiting and diarrhoea
		Healing of childhood diseases
	Instructions given to patients during treatment	Abstinence during traditional treatment of HIV and AIDS
		Stopping breastfeeding
	Adherence to ethics	Maintenance of confidentiality
		Respecting the patient
Negative aspects of traditional healing in the management of HIV and AIDS	Promise of cure	Telling people to stop ARV treatment
		Use of incisions
		Killing of the virus
		Use of emetics and enemas to cleanse the person from the virus.
		Inducing of vomiting and diarrhoea
	Denial of the presence of HIV by the traditional healers	Having an ancestral calling
		Believing that they are bewitched

HIV, human immunodeficiency virus; AIDS, acquired immune deficiency syndrome; ARV, antiretroviral.

This study included 15 key informants, six of whom were women and nine of whom were men. They had all been involved in traditional healing. They ranged in age from 37 to 82 years. See Table 2 for demographic data of key informants.Concurrent with the interviews, one observation was made about traditional healing practices for removing *goni* from a baby. Traditional healing is one of the Vhavenda cultural practices and beliefs that affect HIV and AIDS management, according to the findings. The techniques discussed have both negative and positive aspects as regards to HIV and AIDS management.

**TABLE 2:** Demographic data of the key informants.

Pseudonyms	Age in years	Gender	Traditional practice	Years of practice
Muvhumbe	82	Male	Traditional healer	48
Marubini	67	Female	Traditional healer	39
Ndiambani	57	Male	Traditional healer	25
Ndiimafhi	65	Female	Traditional healer	34
Khakhu	59	Female	Traditional healer	27
Nyakhosi	37	Female	Traditional healer	12
Matodzi	69	Male	Traditional healer	40
Musiwalwo	71	Male	Traditional healer	31
Muvhulawa	75	Male	Traditional healer	54
Nyavele	39	Female	Traditional healer	20
Muvhoni	54	Male	Traditional healer	42
Nyadenga	48	Female	Traditional healer	19
Masindi	80	Male	Traditional healer	49
Makhokha	38	Male	Traditional healer	19
Avhapfani	40	Male	Traditional healer	14

## Positive aspects of traditional healing in the management of HIV and AIDS

Some traditional healers alleged that they heal other opportunistic infections and that they also instruct their clients on things to do whilst on traditional treatment. Traditional healers also maintained confidentiality about the identity of their clients and the treatment used to treat them. The superordinate theme consists of the following three themes: treatment of opportunistic infections, instructions given to patients during treatment and adherence to ethics.

### Treatment of opportunistic infections

The study revealed that traditional healers can treat some opportunistic infections, such as diarrhoea and vomiting, using their own medications as shown to them by their ancestors.

#### Medicines for skin lesions

Traditional healers know how to heal incisions and lesions in a short period of time:

‘During initiation schools, as [*there*] are incisions done to the initiates, I used my own concoction revealed to me by my ancestors to heal the wound at once. The concoction makes the wound dry. It prevents one from infections like HIV as the skin would become intact in a day or two. This also put our boys in a better position as they will heal fast.’ (Marubini, female, 82 years old)

#### Stopping of vomiting and diarrhoea

Muvhumbe alleged that he has treated many people with vomiting and diarrhoea which the hospital failed to treat:

‘If the person come to me having diarrhoea, I can personally stop that. I have helped a lot of people, especially those infected with HIV. They come here vomiting and having diarrhoea, but they are healed after a day or two.’ (Muvhumbe, male, 82 years old)

#### Healing of childhood diseases

According to the findings of the current study, traditional healers can treat *goni*, which is one of the childhood illnesses that is believed to have killed many babies in their neonate and infant stages. Traditional healers also reveal that they can treat a woman who has miscarriaged or lost a baby after birth. It is alleged that they have saved many children by doing that. The following quote from a key informant was found to be appropriate:

‘I purify a woman when a baby dies after birth or through miscarriage. I can also treat *goni* from a child. The process itself is very elaborate. The other problem is that health practitioners says that I am putting men and women at risk of contracting HIV if one of them is HIV-infected. I will not go into detail, but what happens is that I [*take*] blood drawn from the mother and father of the child. Then I mix both husband and wife’s blood through an incision on the navel.’ (Ndiimafhi, female, 65 years old)

### Instructions given to patients during treatment

Traditional healers also give instructions to patients under their care. Clients who are on traditional treatments are expected to abstain from sexual intercourse for the duration of such treatment.

#### Abstinence during traditional treatment of HIV and AIDS

The current study reveals that traditional healing is sacred; that is why traditional healers instruct their clients to abstain from sex until the completion of treatment:

‘I give patient treatment for a bit longer time. Let me say for at least three months. During that period, the person should be abstaining. I do not want to treat the person who is also busy reinfecting himself with HIV. My traditional medicines are sacred.’ (Muvhoni, male, 54 years old)

‘Don’t you know why it is sacred? It is because sexual intercourse is against our traditional healing. You must first finish the treatment. According to Tshivenda, medicine should be taken as sacred.’ (Masindi, male, 80 years old)

#### Stopping breastfeeding

In Vhavenda culture, when a woman is breastfeeding, she is not allowed to take certain traditional treatments as these will have a detrimental effect on the baby or otherwise cause harm to the baby:

‘I know that the child must be breastfed, but I am afraid to give the women treatment of HIV whilst breastfeeding, knowing that the baby will get all the treatment in the breast milk. Another issue will be that there is a possibility that the child can be infected with HIV if the mother is sickly.’ (Muvhoni, male, 54 years old)

### Adherence to ethics

#### Maintenance of confidentiality

Traditional healers also maintain confidentiality when treating their clients. They will never disclose their clients’ HIV status. Traditional healers are trusted by their clients more than healthcare workers are:

‘It’s just that I cannot show you a photo because you may know the person and that poses a problem because it’s a secret. I know my clients will never tell the healthcare workers that he is living with HIV; that is why they come to me in the first place.’ (Marubini, female, 67 years old)

Ndiambani, Musiwalwo and Muvhulawa also share the this view:

‘At present, I am treating the most famous pastor. He is afraid to go to the hospital because they might leak his illness, that he is living with HIV. I cannot reveal his secret, and I will die with it.’ (Ndiambani, male, 57 years old)

‘Without us traditional healers, I am telling it was going to be difficult to control this disease. A lot of people come to us for treatment of HIV and is just that I won’t show you how I treat them, as it will upset my ancestors. You must know that every client has his or her own medication which was revealed to me by my ancestors.’ (Musiwalwo, male, 71 years old)

‘If I was not treating some of the patients with HIV, do you think the government would be able to treat them all? No, it can’t; some of our clients never visited the hospital and some went there but without improvement or no help, and they decided to come to me for help. They are fine now. Unfortunately, I won’t be able to show them to you, but they are good.’ (Muvhulawa, male, 75 years old)

#### Respecting the patient

The study found that traditional healers treat their clients with respect. They do not take advantage of the disease that they are presenting with, namely that they are HIV-positive and that they need help:

‘I treat my clients with respect, even if they come to me being confused, some emaciated and some unable to help themselves, and I must help them to go to the toilet, bathe and feed them. I still respect them; they are my clients, and nothing will make me to not respect them.’ (Makhokha, male, 38 years old)

## Negative aspects of traditional healing in the management of HIV and AIDS

The current study reveals that some traditional healings impacted negatively on the management of HIV and AIDS. This superordinate theme consists of the following two themes: the promise of a cure and denial of the presence of HIV by the traditional healers.

### Promise of cure

Not only are people treated for HIV in the traditional way, but they are also promised a cure. It has been noted that traditional healers believe they can cure HIV, as evidenced by the people they have cured:

‘Hmmm, we traditional healers have a hard job because we heal people even though we are not considered. I have healed several people. In the hospital you cannot do anything; you can only give so many pills. Once they brought me a person who could not even speak; he could not even recognise his children. When the person comes here, you cannot believe it. It’s just that the ancestors do not allow me to show people my medicine. The problem is that when these white people test the medicine, they first want me to show them what I mixed. I cannot show them that.’ (Muvhumbe, male, 82 years old)

#### Telling people to stop treatment

Traditional healers do not want to mix their medicine with Western medicine (ARVs):

‘When someone comes and is sick, I first ask if the person is living with HIV and taking ARVs. Then I treat the person from there. If the person is HIV positive, I first ask them to stop treatment and focus on my treatment alone. I do not want to mix the treatment. After the person is cured, they can go back to the clinic and continue their treatment. I do not want to mix my traditional treatment with Western medicines. I want to prove that my treatment works and that it can cure HIV.’ (Nyadenga, female, 48 years old)

Nyavele and Nyakhosi also instruct their clients to stop using ARVs and focus on their traditional treatment first, so that they can be able to see the effectiveness of traditional medicine in treating HIV and AIDS:

‘Yes, but I instruct them to stop taking ARVs because I do not want to mix my medicines with ARVs or Western medicine. If I mix them, I cannot see whether my client’s treatment has improved or not. Besides, the health authority will pass off my hard work as theirs.’ (Nyavele, female, 39 years old)

‘But if they come to me because they were sick because of ARVs, I usually instructed my clients to stop taking them. I cannot mix my medicines with ARVs.’ (Nyakhosi, female, 37 years old)

#### Use of incisions

‘Another thing done is to remove the so-called dirty blood – infected with HIV – from a client, where blood is removed by doing incisions and the blood is squeezed out of the client, which also predisposes the client to anaemia and infections as the knives used are not sterilised and are used on all clients. [*I*] remove dirty blood from the client’s system; most of the people are killed by the blood which is infected with HIV. I can squeeze that infected blood and then you will be healed.’ (Khakhu, female, 59 years old)

#### Killing the virus

Not only do traditional healers stop people from taking ARVs, but they can also make promises to kill the virus:

‘If it were not for us, I tell you, there would be so many graves because of HIV and AIDS. I have treated many people with HIV and AIDS. I can cure HIV and AIDS. I mean I treat them so they can go back to work. Most of them are incapable of doing anything. You will find that a person does not even know the person who brought them here when they get well. Also, I am not stopping them from taking their antiretroviral treatment.’ (Muvhulawa, male, 75 years old)

‘A person should take medication every month to suppress the virus. This helps. I have many clients out there. They are happy now. Some of them are cured. They no longer tested positive for HIV. If you do the treatment correctly. I will tell you that I can treat HIV. I have my own mixture, but I will never show it to anyone. The ancestors will not allow me to do that.’ (Masindi, male, 80 years old)

#### Use of emetics and enemas to cleanse the person from HIV

Another traditional healer revealed that he uses emetics and enemas to cleanse the person from the virus. The virus should be taken out in the form of vomit or faeces:

‘This is the truth, auntie. I induce vomiting or use an enema because most people who come to me need to be cleaned first before I can commence with any treatment. Remember, with induced vomiting and diarrhoea [*from an enema*], all the dirt will come out and the person will be cleansed from that, and then I will be able to heal the person from HIV.’ (Avhapfani, male, 40 years old)

### Denial of the presence of HIV by traditional healers

Dealing with HIV in Vhembe District is negatively affected because people who are sick or living with HIV believe that they were called by their ancestors to become healers, or they are bewitched. They then undergo training until they become seriously ill and succumb to their illness.

#### Having an ancestral calling

The study found that some of the people believe that they are not infected with HIV, but they have an ancestral calling:

‘Nowadays if you are ill and get emaciated, people think you are suffering from HIV and AIDS, which is not right. Most people are dying because they no longer adhere to our cultural rituals. Instead of the family to arrange for the person to undergo traditional healer’s training, they go to the clinics or hospitals for HIV testing and the person end up dying.’ (Nyavele, female, 39 years old)

#### Believing that they are bewitched

The study found that people believe that they are bewitched and made to believe that they have HIV:

‘You know, some people are not even infected with the HIV, but they are bewitched by their aunt and community witches, as they are jealous of their success. Many people are dying because of lack of knowledge, believing that they have HIV. I have realised that there is no HIV, but people are using this HIV to kill people.’ (Matodzi, male, 69 years old)

## Discussion

This study portrays the negative and positive effects of traditional healing in one of the rural districts of South Africa, Vhembe district in Limpopo province. Due to the traditional nature of the district, most Vhavenda people frequent traditional healers as their first point of care. Other researchers^3^ share the same sentiment that people do not believe in Western medicine. They would rather go to a traditional healer than waste time coming to the health facility. The same views were shared in the study conducted in Bangladesh,^4^ that most of the community members, especially people of low socio-economic status, first approached the traditional healers with their medical problems. Traditional healers are prone to contracting HIV when performing traditional healing because of the incisions they make in the people they are treating, combined with the fact that most of them do not use PPE such as gloves and goggles, as they believe these to be too expensive. The same narrative was shared by Audet et al.^2^ that traditional healers are aware that there are risks associated with exposure during invasive procedures, but that few purchase PPE or use it correctly because it is expensive, and they cannot afford it. If they do not have gloves, they improvise by using a small stick to apply the medicines to the cuts to ensure that there is no touching of the blood with their bare hands.^15^ When a woman loses a child or suffers abortion, both parents’ blood are mixed through an incision on the umbilicus, in order not to lose a child or to abort subsequent pregnancies. Audet et al.^2^ supports this, stating that a widespread practice is the traditional ‘injection’ (described as such by traditional healers, although the wounds could more accurately be described as incisions), in which the healer performs dozens of subcutaneous incisions to rub herbs directly into the bloodied skin.

The current study found that traditional healers believed that they could heal HIV and other related diseases like diarrhoea, as well as childhood illnesses like *goni*, using medicines shown to them by their ancestors, and it is believed that traditional medicines are fresh. Kabiraji and Bhandari practitioners in rural Bangladesh claim to cure all diseases, where these might include HIV and AIDS or any other chronic health difficulties.^4^ This narrative was also shared in a study conducted in Zimbabwe^16^ that stated that a man called Benjamin Burombo is believed to have cured AIDS. In the same study^16^ it was also found that Mrs Chihuri alleged that the patients she treated for HIV, has tested HIV-negative at local medical laboratories.

Women who are nursing are also instructed to stop breastfeeding, as some of the medication is believed to be harmful to nursing mothers. Some traditional healers believe that it is possible for a child to become infected by HIV when the mother takes traditional treatment for HIV. These findings are unique to the current study.

When patients are using traditional treatment, some healers instruct them to stop using the ARVs and to use only their traditional medicine. The reason for this is that they want to show the Department of Health that their medication does indeed work. In the study conducted in Eastern and Southern Africa,^17^ people opt to stop ARVs themselves, without the involvement of traditional healers in favour of traditional medicine believing that they will be cured of HIV.

Another issue found is that traditional healers believe that they can suppress the HIV virus in the same way that ARVs do. Another researcher^16^ also found that some traditional healers in Zimbabwe believe that they can cure AIDS. When people are infected with HIV, they believe that they have an ancestral calling or that they are bewitched, and that is why they are losing weight and getting opportunistic infections like pneumonia and suffering from diarrhoea. Therefore, they do not take ARVs in time but rather go and seek treatment from traditional healers. Audet et al.^18^ also attests to these findings of the current study that people believe that they are being given AIDS through ARVs because they believe they are not sick – and that is why they end up not going to health institutions and why they visit traditional healers. Other traditional healers promise their clients that they can heal HIV. This results in confusion, as they then will not take treatment and end up losing their lives because of delaying the start of ARV treatment. Agreeing with these statements is the study conducted in Maputo, Mozambique,^19^ which found that the use of traditional healers is associated with delayed HIV diagnosis and reduced adherence to antiretroviral therapy (ART).

Some traditional healers reveal that they use emetics and enemas to get their clients ‘to remove all the dirt which is inside their body’ so that they can be able to heal them. They induce vomiting in their clients and induce defecation as a way of treating them. These findings are unique to the current study.

Most traditional healers do not want patients to mix their medications with ARVs, and their patients are thus instructed to stop their ARVs and focus on the traditional treatment first. Another researcher^16^ shared the same narrative that traditional healers who believe that they can cure HIV do not want to mix their treatment with ARVs, fearing that the government will take credit for their work. Traditional healers will never divulge the identity of their clients. They always adhere to the rule of confidentiality and their clients trust them with everything, rather than trusting health workers, who they fear might divulge their information as they are working in groups. Okere et al.^6^ and Rohwer et al.^20^ concur with the statement that resistance to attend health facilities for HIV treatment is due to the lack of confidentiality in the health facilities and the fear of unintentional disclosure of HIV status due to frequent clinic visits. Also sharing the same narrative are findings in the study^3^ conducted in rural Uganda that clients easily confide in their traditional healers rather than in health institutions, as they are afraid of their HIV status being known.

According to the current study, some traditional healers instruct their clients to abstain from sex, as they allege that their medication is sacred and that one cannot engage in sexual intercourse whilst on traditional medication. These findings are unique to the current study.

### Limitations of the study

The study was undertaken only in Vhembe district, Limpopo province, even though it is an ethnographic study. Although this was a comprehensive study, only key informants who are involved in traditional healings and who met the inclusion criteria participated. The authors explained the steps taken to ensure the study’s credibility because the Vhembe district includes people from Vhavenda areas, as well as other parts that have relations with the Vhavenda people of the district. Along with the interviews, only one observation was undertaken to acquire data, which is a drawback because ethnographic studies rely largely on observations. Due to the confidential nature of traditional healings, the researcher was denied access to witnessing other rituals, such as purifying a woman who just lost a baby or pregnancy, but was allowed to witness the removal of *goni* from a child who was sick. Because some cultural events are considered sacred and confidential, the researcher were not allowed to observe them.

### Recommendations

There is a need for nurturing common understanding and regard amongst all the traditional healers involved in HIV and AIDS management. Collaborations of traditional healers, health professionals and the Department of Health in the management of HIV and AIDS and other related diseases are of paramount importance to reducing new HIV infections and associated deaths. Effective, two-way referral systems should be in place for HIV testing, early diagnosis of HIV infections and the management of opportunistic infections. Traditional healers should be given slots on the local radio stations and should also be given time to go to school to teach young children about the importance of traditional healing in managing HIV and AIDS.

## Conclusion

Based on the findings, the researcher recommend that activities with a high risk of HIV infection be modified to lower the risk, whilst continuing to support positive practices that reduce HIV mortality, such as diarrhoea management. Traditional healers should be trained in the treatment of HIV and AIDS, use and wearing of PPE and infection prevention control so that they are able to treat people with HIV without compromising their health and delaying their treatment. Both the Ministry of Health and traditional healers should work together to achieve the proper management of HIV and AIDS by both health institutions and traditional healers, with neither misleading the other due to a lack of knowledge on the part of the traditional healers and their clients. The findings will contribute to the body of knowledge in the field of health and will have an impact on HIV and AIDS policy change. The findings will also have an impact on HIV and AIDS management practices by providing contextualised, culturally relevant information and approaches. This will also help with HIV prevention, treatment and care being re-Africanised.

## References

[1] Global AI. Update. Confronting inequalities-lessons for pandemic responses from 40 years of AIDS [homepage on the Internet]. Geneva: UNAIDS Joint United Nations Programme on HIV/AIDS; 2021 [cited 2022 Apr 05]. Available from: https://www.unaids.org/en/resources/documents/2021/2021-global-aids-update

[2] Audet CM, Shepherd BE, Aliyu MH, Moshabela M, Pettapiece-Phillips MJ, Wagner RG. Healer-led vs. clinician-led training to improve personal protective equipment use among traditional healers in South Africa: A randomized controlled trial protocol. Global Health Action. 2021;14(1):1898131. 10.1080/16549716.2021.1898131 33797347 PMC8023590

[3] Broderick K, Ponticiello M, Nabukalu D, et al. Shortening ‘the road’ to improve engagement with HIV testing resources: A qualitative study among stakeholders in rural Uganda. AIDS Patient Care STDs. 2021;35(2):56–62. 10.1089/apc.2020.0235 33471578 PMC7885900

[4] Haque M, Chowdhury AB, Shahjahan M, Harun M, Dostogir G. Traditional healing practices in rural Bangladesh: A qualitative investigation. BMC Complement Altern Med. 2018;18(1):1–5. 10.1186/s12906-018-2129-5 29448941 PMC5815193

[5] Stats SA. Statistical Release P0302. Mid-year population estimates 2020. Pretoria: Department of Statistics South Africa; 2021.

[6] Okere NE, Sambu V, Ndungile Y, et al. The Shinyanga patient: A patient’s journey through HIV treatment cascade in rural Tanzania. Int J Environ Res Public Health. 2021;18(16):8418. 10.3390/ijerph18168418 34444166 PMC8393654

[7] Privitera GJ. Research methods for the behavioral sciences. 2nd ed. New York, NY: Sage; 2018.

[8] Vhembe District Health Information System. Annual report. Pretoria: Government Printers; 2019.

[9] Joubert G, Ehrlich R, Katzenellenbogen J, Karim SA. Epidemiology: A research manual for South Africa. Pretoria: Oxford University Press Southern Africa; 2014.

[10] Gray JR, Grove SK, Sutherland S. Practice of nursing research: Appraisal, synthesis, and generation of evidence. 8th ed. St. Louis, MO: Elsevier; 2017.

[11] Bryman A, Bell E. Research methodology: Business and management contexts. Cape Town: Oxford University Press Southern Africa; 2014.

[12] Brink H, Van der Walt C, Van Rensburg G. Fundamentals of research methodology for health care professionals. 4th ed. Cape Town: Juta and Company Ltd; 2018.

[13] Polit DF, Beck CT. Nursing research: Generating and assessing evidence for nursing practice. 10th ed. Philadelphia, PA: Lippincott Williams & Wilkins; 2017.

[14] Creswell JW, Creswell JD. Research design: Qualitative, quantitative, and mixed methods approaches. New York, NY: Sage; 2017.

[15] Audet CM, Gobbo E, Sack DE, et al. Traditional healers use of personal protective equipment: A qualitative study in rural South Africa. BMC Health Serv Res. 2020;20(1):1–6. 10.1186/s12913-020-05515-9 PMC7362457 32669101

[16] Shoko T. Traditional herbal medicine and healing in Zimbabwe. J Tradit Med Clin Naturop. 2018;7(1):1–3. 10.4172/2573-4555.1000254

[17] Moshabela M, Bukenya D, Darong G, et al. Traditional healers, faith healers and medical practitioners: The contribution of medical pluralism to bottlenecks along the cascade of care for HIV/AIDS in Eastern and Southern Africa. Sexually Transmitted Infections. 2017;93(Suppl 3):e052974. 10.1136/sextrans-2016-052974 28736393 PMC5739844

[18] Audet CM, Salato J, Vermund SH, Amico KR. Adapting an adherence support workers intervention: Engaging traditional healers as adherence partners for persons enrolled in HIV care and treatment in rural Mozambique. Implementation Sci. 2017;12(1):1–2. 10.1186/s13012-017-0582-z PMC5390357 28407813

[19] Sundararajan R, Langa PV, Morshed T, Manuel S. Traditional healers as client advocates in the HIV-endemic region of Maputo, Mozambique: Results from a qualitative study. SAHARA J. 2021;18(1):77–85. 10.1080/17290376.2021.1909492 33902401 PMC8081305

[20] Rohwer A, Hendricks L, Oliver S, Garner P. Testing for saturation in qualitative evidence syntheses: An update of HIV adherence in Africa. PLoS One. 2021;16(10):e0258352. 10.1371/journal.pone.0258352 34665831 PMC8525762

